# Complete Androgen Insensitivity Syndrome: A Rare Case of Prenatal Diagnosis

**DOI:** 10.1055/s-0041-1735986

**Published:** 2021-10-20

**Authors:** Maria Liz Coelho, Elisa Soares, Marília Freixo, Pedro Brandão, Carla Marinho, Juliana Rocha, Graça Rodrigues

**Affiliations:** 1 Department of Gynecology and Obstetrics, Centro Hospitalar Tâmega e Sousa, Porto, Portugal

**Keywords:** prenatal diagnosis, prenatal genetic counseling, androgen insensitivity syndrome, disorder of sex development, 46, XY, androgen receptor, diagnóstico pré-natal, aconselhamento genético pré-natal, síndrome de insensibilidade aos androgênios, distúrbios do desenvolvimento sexual, 46, XY, receptor de androgênios

## Abstract

With the widespread uptake of noninvasive prenatal testing (NIPT), a larger cohort of women has access to fetal chromosomal sex, which increases the potential to identify prenatal sex discordance. The prenatal diagnosis of androgen insensitivity syndrome (AIS) is an incidental and rare finding. We wish to present the diagnosis of a prenatal index case after NIPT of cell-free fetal DNA and mismatch between fetal sex and ultrasound phenotype. In this particular case, the molecular analysis of the androgen receptor (AR) gene showed the presence of a pathogenic mutation, not previously reported, consistent with complete androgen insensitivity syndrome. Carrier testing for the mother revealed the presence of the same variant, confirming maternal hemizygous inheritance. Identification of the molecular basis of these genetic conditions enables the preimplantation or prenatal diagnosis in future pregnancies.

## Resumo

Com a utilização generalizada de testes pré-natais não invasivos (TPNIs), uma crescente porção de mulheres tem acesso ao sexo cromossômico fetal, o que aumenta o potencial para identificar discordância sexual pré-natal. O diagnóstico pré-natal da síndrome de insensibilidade androgénica é um achado incidental e raro. Pretendemos apresentar um caso índice de diagnóstico pré-natal por meio de DNA fetal livre e incompatibilidade entre sexo fetal e fenótipo ecográfico. Neste caso particular, a análise molecular do gene do receptor de andrógenios (RA) revelou a presença de uma mutação patogênica, não relatada anteriormente, consistente com a síndrome de insensibilidade completa aos androgênios. A mãe revelou ser portadora da mesma variante, confirmando a hereditariedade hemizigótica. A identificação da base genética permite o diagnóstico pré-implantação ou pré-natal em futuras gestações.

## Introduction

With the widespread uptake of noninvasive prenatal testing, a larger cohort of women has access to fetal DNA information. Prenatal genetic tests can disclose fetal chromosomal sex, even though these tests were originally designated to diagnose aneuploidies. Therefore, the potential to identify prenatal sex discordance is likely to increase. The prenatal diagnosis of androgen insensitivity syndrome is an incidental and rare finding. It has been reported previously with a discrepancy between the fetal phenotype and the results of sex chromosome analysis following invasive prenatal tests. 1 2 Androgen insensitivity syndrome (AIS; Online Mendelian Inheritance in Man [OMIM]: 300068) is an X-linked recessive genetic disorder with an XY karyotype, caused by androgen receptor defects. 1

Androgen insensitivity syndrome consists of varying degrees of impairment in the function of the androgen receptor (AR) gene and, subsequently, androgen resistance; it can be subdivided into three phenotypes: complete androgen insensitivity syndrome (CAIS), in which the patients exhibit normal or near-normal female phenotype; partial androgen insensitivity syndrome (PAIS), in which the patients present with an ambiguous phenotype; and mild androgen insensitivity syndrome (MAIS), in which the patients have predominantly male phenotype. 3

Complete androgen insensitivity syndrome, the most frequent manifestation of AIS, has a prevalence of 1 in 20,000 to 64,000 live male births. 3 It was first described by Morris 4 in 1953, and it is typically diagnosed when a phenotypic female presents with testicle-like masses in the inguinal region or with primary amenorrhea in adolescence. The syndrome is characterized by female external genitalia, testicles in the abdomen location or descended, absent mullerian duct derivatives, and 46, XY karyotype. 3

We wish to present this rare prenatal diagnosis of CAIS, which was an incidental finding after noninvasive prenatal testing (NIPT) of cell-free fetal DNA.

## Case Report

The fetus of a 34-year-old healthy primigravida, with no family history of congenital anomalies, was found to have high risk for trisomy 21 (1:220), based on the combined screening results of the early first trimester (pregnancy-associated plasma protein A and free human β-chorionic gonadotropin levels at 10 weeks plus nuchal translucency and nasal bone assessment at 13 weeks).

The patient underwent NIPT (TOMORROW prenatal test, Unilabs, Porto, Portugal), which revealed a low risk for trisomies 21, 18 and 13. The sex chromosome analysis indicated a probability of XY of 99%. The fetal fraction analysis was performed using the VeriSeq NIPT Analysis Software (16 Samples) (Illumina, Inc., San Diego, CA, United States), and was reported at 12%.

**Fig. 1:**
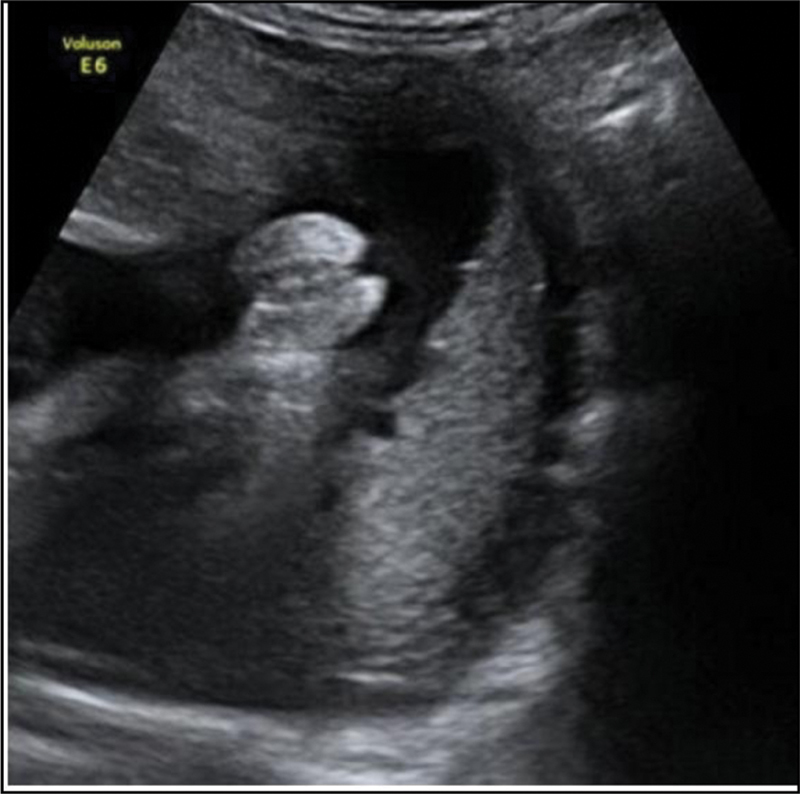
Identification of female external genitalia on the midtrimester ultrasound scan.

The fetus showed no structural abnormalities on the midtrimester ultrasound scan; however, female external genitalia were identified ( Fig. 1 ).

Due to the mismatch between fetal sex and ultrasound phenotype, amniocentesis was performed at 22 weeks of gestation and subsequently sent to quantitative fluorescent polymerase chain reaction (QF-PCR) analysis and a comparative genomic hybridization (CGH) microarray study.

The QF-PCR showed a profile consistent with a male fetus disomic for 13, 18, and 21. The CGH microarray study showed no chromosomal imbalances. The AR gene molecular analysis (NextSeq, Illumina, Inc.) showed the presence of a pathogenic mutation, c.1234del(Ala412 > Argfs67), in hemizygosity, consistent with CAIS. This mutation, which has not been previously reported in the literature, originates a frameshift and a premature stop codon, resulting in a truncated dysfunctional protein. Studies for the sex-determining region Y (SRY) gene and sequencing of the 5-α reductase gene were normal.

The couple received genetic and psychological counseling and proceeded with the pregnancy. At 40 weeks of pregnancy, the patient delivered a phenotypically normal female newborn. Given that this is an X-linked recessive condition, carrier testing for the mother was undertaken, and the AR gene sequencing revealed the presence of the same variant, c.1234del(Ala412 > Argfs67), confirming the maternal hemizygous inheritance.

## Discussion

In the case herein presented, the ultrasonography and cytogenetic analysis showed discordance between chromosomal and phenotypic sex, suggesting the diagnosis of CAIS, 1 which is caused by an AR dysfunction, leading to complete androgen resistance. The AR gene consists of 910 to 919 amino acids, and it is located on the long arm of the X chromosome (Xq11–12). 5

To date, more than one thousand different mutations have been reported affecting the splicing of the AR RNA (the Androgen Receptor Gene Mutations Database World Wide Web Server, http://androgendb.mcgill.ca/ ): ∼ 70% of them are maternally inherited, while the remaining 30% are de novo mutations, either germline or somatic. 6

The mutation detected in this particular case has never been described before in the literature, and the prenatal diagnosis of CAIS in an index case has been reported only a few times. 1 7 8

Although CAIS is the most common form, resulting in feminization of a 46, XY fetus, there are other syndromes one must consider: SRY gene mutations, 5-α reductase deficiency, Swyer syndrome, 17-α-hydroxylase deficiency, and Smith-Lemli-Optiz syndrome. 9

Without fortuitous prenatal testing, the sex discordance would have gone unidentified until puberty or later. The management of CAIS should depend on a multidisciplinary team, including endocrinology, urology, gynecology and clinical psychology. 3

The current recommendation for CAIS patients is to perform bilateral gonadectomy to avoid age-related malignancy (< 1% in prepubertal subjects versus > 20% in late adulthood). 10 The surgery is usually deferred until after puberty, so that patients may profit from the aromatized androgens, resulting in spontaneous development of estrogen-dependent secondary sexual characteristics. 3

Correct identification of the molecular basis of these genetic conditions is of high importance, as it enables the preimplantation or prenatal diagnosis in future pregnancies. As an X-linked recessive disease, there is a likelihood of 50% that an XY offspring will be affected and that an XX offspring will be an asymptomatic carrier.

The NIPT for fetal sex determination using cell-free fetal DNA has a high concordance rate of over 99.4%. 11 With its increasing availability, as well as high-resolution ultrasound, more disorders of sex development will be identified prenatally. Prenatal molecular analysis of the AR gene is available and leads to definitive diagnosis.
